# Image local structure information learning for fine-grained visual classification

**DOI:** 10.1038/s41598-022-23835-0

**Published:** 2022-11-10

**Authors:** Jin Lu, Weichuan Zhang, Yali Zhao, Changming Sun

**Affiliations:** 1 School of Electronic Information and Artificial Intelligence, Shaanxi University of Science & Technology, Xi’an, 710021 China; 2 The Institute for Integrated and Intelligent Systems, Griffith University, Brisbane, QLD Australia; 3 School of Electronics and Information, Xi’an Polytechnic University, Xi’an, 710000 China; 4 CSIRO Data61, PO Box 76, Epping, NSW 1710 Australia

**Keywords:** Computer science, Information technology

## Abstract

Learning discriminative visual patterns from image local salient regions is widely used for fine-grained visual classification (FGVC) tasks such as plant or animal species classification. A large number of complex networks have been designed for learning discriminative feature representations. In this paper, we propose a novel local structure information (LSI) learning method for FGVC. Firstly, we indicate that the existing FGVC methods have not properly considered how to extract LSI from an input image for FGVC. Then an LSI extraction technique is introduced which has the ability to properly depict the properties of different local structure features in images. Secondly, a novel LSI learning module is proposed to be added into a given backbone network for enhancing the ability of the network to find salient regions. Thirdly, extensive experiments show that our proposed method achieves better performance on six image datasets. Particularly, the proposed method performs far better on datasets with a limited number of images.

## Introduction

It is well known that object classification is essential and important in computer vision and image processing. For the past few years, sustained and stable progress has been gotten in fine-grained visual classification (FGVC). On one hand, many deep neural networks^1–8^ with improved learning ability to recognize the subtle differences between highly similar objects have been designed. On the other hand, amounts of fine-grained image datasets, including bird species^9^, car^10^, aircraft^11^, and ultra-fine-grained (UFG)^12^, are collected by domain experts. In these datasets, complex rules is used for measuring the accuracy of object classification methods, and also benefit for improving better algorithms.

The key step of FGVC is to learn discriminative information from salient regions. The existing FGVC methods fall into two groups. The methods in the first group^13^ intend to optimize the neural network structure for learning discriminative information from salient regions. The methods in the second group^14^ try to locate the salient regions by a bounding box or part annotations mechanism^15–17^ and then perform object classification using the discriminative information from the selected regions.

As we know, extracting (local structure information) LSI from each input image is the basic step of FGVC. At present, a lots of LSI extraction techniques such as first- and second-order derivative^18,19^ have been proposed. Moreover, image data augmentation techniques is widely used to increase the efficiency of LSI extraction for better finding the discriminative regions and improving the performance of FGVC, including^20^, image rotation^21^, image flip^5,7,22^, and image affine transformations^23^. However, within the scope of our investigations, no one has systematically studied how to properly depict different local structure features (e.g., edge, corner, and blob) in each input image for object classification in the field of FGVC. The reason is that they have not considered how to properly extract LSI from each input image and also have not considered the properties of different types of image local structure features and the differences among them. For example, Feng et al.^21^ intend to use original image and rotated image (e.g., rotating the original image counterclockwise by $\pi$/2, $\pi$, and 3$\pi$/2) for enhancing the ability for feature learning. However, it is recently^24,25^ demonstrated that the LSI between the image and the image rotated by $\pi$ are the same.

In this paper, the first- and second-order directional derivative^25–35^ of image local structural features are utilized to investigate the properties of the features which also enable us to study the existing LSI extraction, image data augmentation, and description of local structure feature techniques. Our research indicates that the existing image data augmentation techniques (e.g., lighting changes^36^, colorizing image^20^, and image affine transformations^23^) have a great impact on the performance of FGVC. If the extraction of LSI and the description of local structure features from each input image are not carefully considered in the existing image date augmentation techniques, they cannot efficiently enhance the ability of a network to extract LSI from each input image which can cause the stability issue of FGVC or even weaken the performance of FGVC. The aforementioned phenomena are more likely to happen under unsupervised conditions. Meanwhile, the first- and second-order directional derivatives of edge, corner, and blob indicate that it is necessary for us to extract LSI of local structure features along multiple filter orientations. Only in this way, can we properly obtain the LSI of different local structure features.

In this work, we propose a novel LSI learning method for FGVC. The idea of extracting image LSI along multiple filter orientations and the idea of attention enhancement mechanism (AEM)^37^ are combined to efficiently extract LSI from each input image and localize salient regions automatically for FGVC. Besides adequately extracting LSI from each input image, no additional auxiliary conditions is required by our proposed method to prevent overfitting and noise influence. Furthermore, the overall structure information of objects has been considered in our method.

The main contributions of our proposed method comprise three aspects. Firstly, our unique way of LSI extraction from an input image is illustrated by an example of the first- and second-order directional derivative based LSI extraction of local structure features. Furthermore, the extracted LSI has the ability to properly depict the complete local structural features in images. Secondly, a novel LSI learning method requiring no additional object notation is proposed for FGVC. Thirdly, the proposed method outperforms eight state-of-the-art FGVC methods in five standard image datasets (i.e., UFG^12^, flower^38^, bird species^9^, car^10^, and aircraft^11^).

This paper is organized as follows. In section “Related work”, the problem of FGVC and the existing FGVC methods are briefly introduced. In section “Proposed method”, we propose a novel LSI learning method after showing how to extract LSI from an input image. In section “Experiments”, we demonstrate the performance of our proposed method on six standard datasets by comparing with the eight existing benchmark methods.

## Related work

There are two aspects of FGVC problem, the first is how to make a given network identify discriminative regions, and the second is how to learn the structure of objects. The existing FGVC methods can be roughly divided into two categories. In the first category of methods^15–17^, first the salient regions are located, then FGVC is performed based on the structure information of objected from the selected regions. It is worth to note that these methods^15–17^ usually spend so much time in collecting annotations according to a bounding box or part annotations mechanism.

In the second category of methods^3–8,22,39^, the salient regions is determined by optimizing the neural network structure. Fu et al.^39^ proposed an attention mechanism to locate the salient regions, then features are learned in the selected regions by using multi-scale technique. Yang et al.^4^ proposed a multi-agent learning mechanism to identify information regions, then the selected regions was carefully checked for FGVC. Chen et al.^5^ proposed a destruction and construction learning (DCL) mechanism, which had better ability to learning discriminative regions and features. Zhou et al.^7^ showed that identifying holistic structure of different objects in each input image was benefit for locating salient regions. Min et al.^8^ enlarged bilinear pooling technique^40^ to a multi-object matrix normalization (MOMN) method, which has the ability to simultaneously regularize a second-order representation based on square-root, low-rank and sparsity.

Additionally, image data augmentation techniques are considered as good assistant of FGVC. The image data augmentation have the function of increasing the diversity and the amount of training data, which help to lower the chance of network overfitting and improve the classification performance. The image data augmentation techniques can be classified into two groups. The first group is manual image data augmentation techniques, including image geometric transformations, flipping, colorizing image, cropping, rotation, noise injection, and mixing images. The second group is automatic augmentation^41^, including auto augmentation learning^42^ and random erasing data augmentation^43^.

## Proposed method

**Figure 1:**
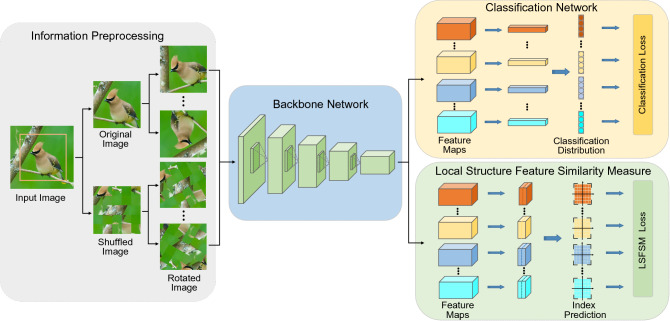
The overall pipeline of our proposed LSI learning framework. (1) Information preprocessing: rotate the input and shuffled images. (2) Backbone classification network: extract the basic feature maps. (3) Classification network: classify images into fine-grained categories. (4) LSFSM: measure local structure features similarity of different images.

In this section, we firstly present the way of properly extracting LSI from an input image and secondly propose a novel LSI learning method for FGVC. Figure 1 shows the overall pipeline of our propose LSI learning framework, including four modules as LSI preprocessing, backbone classification network, classification network, and local structure feature similarity measure (LSFSM).

### LSI extraction

It is well known that the accuracy of LSI has great influence over subsequent tasks of an input image in computer vision and image processing. As the basic structural feature of an image, image corner and edge are generally detected by using the first-order derivatives^25,27,44^, and blob are generally detected by using the second-order derivatives^45^. Next, examples of these three basic structural features detection are used to show our way to extract LSI from an input image, in which both the scale factor and the anisotropic factor are set to $\sqrt{1.5}$.

**Figure 2:**
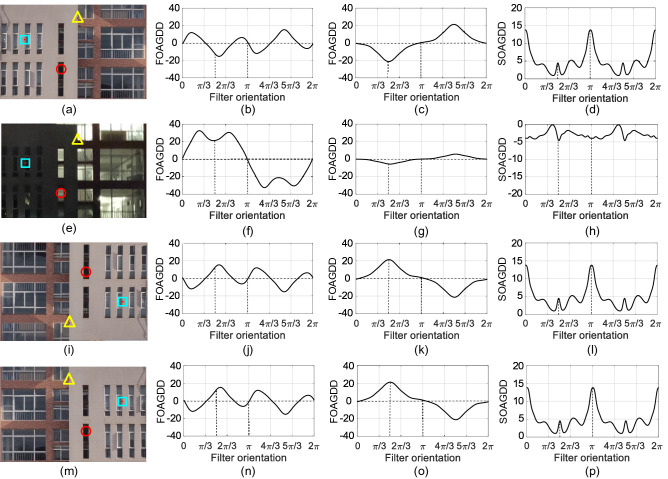
Examples of the FOAGDDs at a corner (marked by ‘$\Delta$’)and an edge point (marked by ’$\Box$’) and the SOAGDDs at a blob (marked by ‘$\bigcirc$’) at the same location under different imaging conditions.

Figure 2a is the test image ’Building’, where a corner is indicated as a ‘$\Delta$’, an edge point is indicated as a ‘$\Box$’, and a blob is indicated as a ‘$\bigcirc$’. Figure 2b–d are the FOAGDD of a T-type corner, the FOAGDD of the step edge, and the SOAGDD of the blob along different filter orientation, respectively. It can be seen from Fig. 2b and c that the variation of the directional derivative along filter orientation from 0 to 2$\pi$ is different for T-type corner and step edge. That is, the directional derivative of the T-type corner has three local maxima and three local minima, and the directional derivative of the step edge has only one local maximum and one local minimum. Figure 2b and c also indicate that the FOAGDDs at horizontal and vertical filter orientations cannot distinguish the corner from the step edge, which can be explained by the FOAGDD representations of corners and edges^25,28^. This phenomena reminds us that the LSI of an input image should be extracted from multiple filter orientations. Figure 2e is the test image ’Building’ with lighting change. Figure 2f–h are the FOAGDD of the corner, the FOAGDD of the step edge, and the SOAGDD of the blob, respectively. Figure 2f–h clearly show that, the FOAGDD of the corner are larger in many filter orientations, by contrast, the FOAGDD of the edge and the SOAGDD of the blob are smaller in many filter orientations. Therefore, lightning condition has great impact on the LSI extraction and the subsequent tasks such as the description and classification of different local structural features.

Meanwhile, image rotation^21^ or image horizontal flip^5,7^ is a widely used operation in image data augmentation for FGVC. After rotating the original image counterclockwise by $\pi$ as illustrated in Fig. 2i, it can be seen from Fig. 2j–l that the absolute first-order directional derivative of the corner and edge and the second-order directional derivative of the blob are equal to the values of the corresponding positions on the original image as shown in Fig. 2b–d. After horizontally flipping the original image as illustrated in Fig. 2m, it can be seen from Fig. 2n–p that the absolute first-order directional derivative of the corner and edge and the second-order directional derivative of the blob are equal to the values of the corresponding positions on the original image.

Based on the above examples, we can find that some image data augmentation operations can make the LSI of the local structure features prominent and make it easy for classification, while some image data augmentation operations will make the LSI of the local structure features less prominent and make it difficult for classification, and some image data operations have no effect on LSI extraction. Furthermore, multi-scale techniques^16,39^ also have been widely used for enhancing the LSI extraction and performing FGVC. Zhang and Sun^25,32^ revealed that the existing multi-scale techniques can only efficiently enhance the LSI extraction along the established filtering orientation of backbone networks. The key of LSI extraction from an input image is to extract LSI along multiple filter orientations. The reason is that only by extracting LSI of each input image along multiple orientations, can the properties of different local structure features be properly depicted. It means that when performing FGVC, we need to process the extracted local structure information of an input image in different filter orientations at the same time. Only in this manner can we accurately extract sufficient LSI from each input image for analyzing the properties of different salient regions and performing more effective FGVC.

### Information preprocessing

AEM^37^ is an efficient way to make a network concentrated on learning local salient contents. We will extend the AEM from one-dimensional signal to two-dimensional signal for FGVC. For an input image *I*, we first establish its corresponding Cartesian coordinates based on the central pixel of the image. The input image is partitioned into $N\times N$ sub-image blocks *B* *i*, *j* where *i* ($-\lfloor \frac{N}{2} \rfloor \le i \le \lfloor \frac{N}{2} \rfloor$) and *j* ($-\lfloor \frac{N}{2} \rfloor \le j \le \lfloor \frac{N}{2} \rfloor$) represent the horizontal and vertical indices respectively. Then each sub-image block *B*(*i*, *j*) is placed in the image with uniform distribution. The shuffled image is denoted as *S*. It is worth to note that the shuffled image in AEM will make the network concentrate on local salient regions. However, AEM will make the network ignore the overall structure information of object.

**Figure 3:**
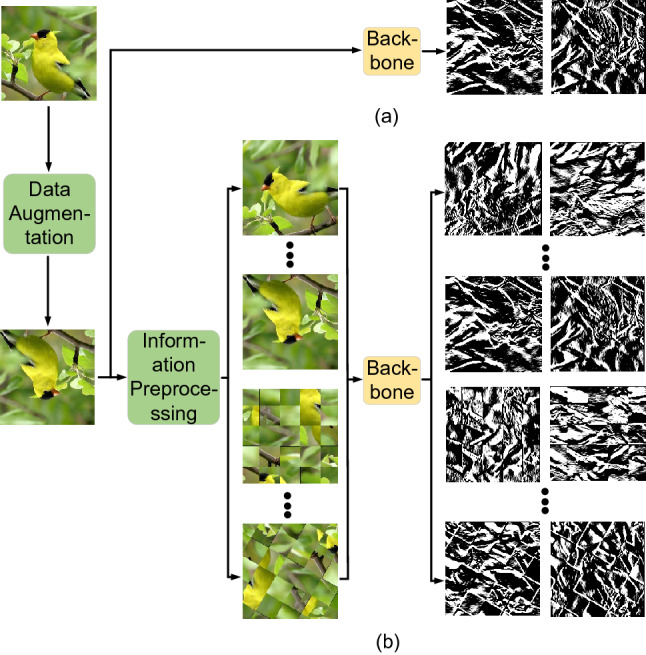
Examples of the LSI extraction. (**a**) LSI extraction of the existing image data augmentation technique. (**b**) LSI extraction of our proposed information preprocessing.

We rotate both the original image *I* and shuffled image *S* in interval $\frac{\pi }{K}$ in the range of $[0,\frac{(K-1)\pi }{K}]$, which enhance the ability of the network on learning the salient local regions of objects and the overall structure of objects. Then a series of rotated original images $I_k$ ($k= 1,2,\dots$,$K-1$) and rotated shuffled images $S_k$ ($k=1,2,\dots$,$K-1$) are fed into the backbone network for training. Figure 3 is an example of a backbone network for extracting the first-order intensity variation information of each input image. It can be seen from Fig. 3a that, with the existing image date augmentation technique, only the LSI along a pair of orthogonal orientations is extracted from each input image in each epoch. By contrast, with our operation, the LSI along 4$(K-1)$ orientations is extracted from each input image in each epoch, as shown in Fig. 3b. In this way, the network has a high chance to obtain enough LSI from each input image for feature learning. This is impossible for the existing state-of-the-art FGVC methods^1–5,7,8^, as they have not considered how to use LSI for accurately depicting local structure features and performing FGVC. Experimental comparisons illustrate that our method performs far better when the number of trainning images in the dataset is limited.

### Classification network

Commonality always exists among the objects in different images of the same category. According to the information preprocessing module, the rotated original images $I_k$ ($k=1,2,\ldots$,$K_1$) and the rotated shuffled images $S_d$ ($d=1,2,\ldots$,$K_2$) are transformed from the input image *I* for our method. After that, the set $\{I_1$,$\ldots$,$I_{K_1}$,$S_1$,$\ldots$,$S_{K_2},\varvec{l}\}$ is training, where *l* is the corresponding ground truth one-vs-all label indicating fine-grained categories. Image group $\{I_1$,$\ldots$,$I_{K_1}$,$S_1$,$\ldots$,$S_{K_2}\}$ is sent to the backbone network to obtain the corresponding feature maps. Next, an adaptive average pooling layer and a fully connected layer in classification network are used to process the feature maps to obtain the classification distribution $\{\varphi (I_1)$,$\ldots$,$\varphi (I_{K_1})$, $\varphi (S_1)$,$\ldots$,$\varphi (S_{K_2})\}$. In this way, the classification loss $L_{c}$ is defined as

1 $$\begin{aligned} \begin{aligned} {L_{c}} = - \sum \limits _{I \in \mathrm{C}} \bigg ({\sum \limits _{k = 1}^{{K_1}} {{{\varvec{l}}}\cdot \log (\varphi ({I_k}))} } + {\sum \limits _{d = 1}^{{K_2}} {{{\varvec{l}}}\cdot \log (\varphi ({S_d}))} }\bigg ), \end{aligned} \end{aligned}$$

where *C* represents the image set for training.

### Local structure feature similarity measure

It is worth to note that the aforementioned classification network is to perform FGVC by learning holistic and local information of objects. Inspired by^46^, similarity measurement of local regions among different images are introduced to make the network learn more LSI of objects for better FGVC.

It is worth to note that the positions of the sub-images have changed after the original image is rotated or shuffled. It is necessary for us to give a new index for the rotated or shuffled image in the information preprocessing module. For each rotated original image $I_k$ ($k=1,2$,$\ldots$,$K_1$), its corresponding index (*u*, *v*) of sub-image block $B_k(u,v)$ can be obtained by the product of the index (*i*, *j*) of the original image block *B*(*i*, *j*) and a rotation matrix ${\mathbf {R}}_{k}$

2 $$\begin{aligned} \begin{aligned} {[u,v]}=&{[i,j]}{\mathbf {R}}_{k},\\ {\mathbf {R}}_{k}=&\left[ \begin{array}{cc} \cos (\frac{(k-1)\pi }{K_1})&{}-\sin (\frac{(k-1)\pi }{K_1})\\ \sin (\frac{(k-1)\pi }{K_1})&{}\cos (\frac{(k-1)\pi }{K_1}) \end{array} \right] . \end{aligned}\end{aligned}$$

Given a sub-image block *B*(*i*, *j*) of the original image *I*, the average gray value of the sub-image block *B*(*i*, *j*) is compared with the average gray value of each sub-image block $S_1(m,n)$ of the shuffled image $S_1$. The index (*i*, *j*) of the sub-image block *B*(*i*, *j*) is assigned to the index (*m*, *n*) of the sub-image block $S_1(m,n)$ when the average gray value of the two sub-images is the closest. In this way, the index (*m*, *n*) of each sub-image block $S_1(m,n)$ is obtained. Meanwhile, the index (*p*, *q*) of the sub-image block $S_d(p,q)$ of the rotated shuffled image $S_d$ ($d=1,2$,$\ldots$,$K_2$) can be obtained by the product of the index (*m*, *n*) of the shuffled image block $S_1(m,n)$ and the rotation matrix ${\mathbf {R}}_{k}$ using Eq. (2).

In this module, the indices of $\{I_1$,$\ldots$,$I_{K_1}$,$S_1$,$\ldots$,$S_{K_2}\}$ are used as labels. This group of images $\{I_1$,$\ldots$,$I_{K_1}$,$S_1$,$\ldots$,$S_{K_2}\}$ are sent to the backbone network, and their corresponding feature maps are obtained. For each feature map, it is processed by a $1\times 1$ convolution layer, an activation function Tanh, an average pooling layer, reshape, and permuting the array dimensions for obtaining the prediction result of the index of each image block. The results of index prediction of the rotated original image and the rotated shuffled image are denoted as $(\tau _{k}(u),\tau _{k}(v))$ ($k=1,2,\ldots$,$K_1$) and $(\varepsilon _{d}(p),\varepsilon _{d}(q))$ ($d=1,2,\ldots$,$K_2$) respectively. Then the Euclidean distance is used to measure the similarity of local features by calculating the difference between the index labels of input images and their corresponding index prediction results.

3 $$\begin{aligned} \begin{aligned}{}&{L_{sm}}=&\sum \limits _{k = 1}^{{K_1}} {\sum \limits _{u = - \lfloor \frac{N}{2}\rfloor }^{\lfloor \frac{N}{2}\rfloor } {\sum \limits _{v = - \lfloor \frac{N}{2}\rfloor }^{\lfloor \frac{N}{2}\rfloor } {\sqrt{{{(\tau _{k}(u) - u)^2+(\tau _{k}(v) - v)^2}}} } } } +&\sum \limits _{d = 1}^{{K_2}} {\sum \limits _{p = - \lfloor \frac{N}{2}\rfloor }^{\lfloor \frac{N}{2}\rfloor } {\sum \limits _{q = -\lfloor \frac{N}{2}\rfloor }^{\lfloor \frac{N}{2}\rfloor } {\sqrt{{{(\varepsilon _{d}(p) - p)^2 + (\varepsilon _{d}(q) - q)^2}}} } } }. \end{aligned} \end{aligned}$$

Finally, we show the pseudo code of our proposed LSI learning based FGVC algorithm.
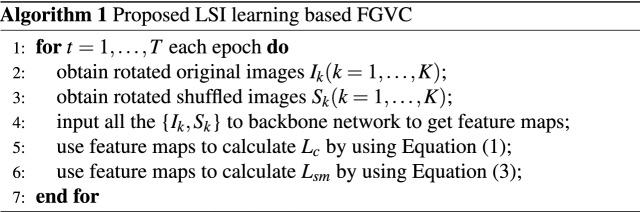


## Experiments

In this section, firstly, the standard datasets, including UFG image datasets^12^,CUB-200-2011 (CUB)^9^, Stanford Cars (CAR)^10^, FGVC-Aircraft (AIR)^11^, Oxford Flower (FLO)^38^, and plant disease (PD)^47^, and experiment settings we used in experiments are introduced. Secondly, the relationship between information preprocessing and the proposed method is illustrate. Thirdly, the performances of the proposed LSI learning method and eight state-of-the-art methods, including ResNet-50^2^,VGG-16^1^, NTS-Net^4^, fast-MPN-Cov^3^, DCL^5^, Cross-X^6^, MOMN^8^, and ACNet^22^, are compared according to several experiments. The codes of these benchmark methods are obtained from their authors.

### Experiment setting

The proposed method and aforementioned state-of-the-art benchmark methods are applied to the six image datasets then their classification performance are compared. Moreover, we emphasize that in our experiments the only annotation used for training is the classification labels of the image datasets. The proposed method is implemented in Pytorch using a 3.50 GHz CPU with 64 GB memory and four NVIDIA Geforce GTX TITAN X with 12 GB memory.

The UFG datasets^12^ include a soybean dataset and a cotton dataset. The cotton dataset contains 80 cotton leaf categories with 3 training images per category. It also includes 240 images as testing data. The soybean dataset contains 1200 images of 200 cultivars of soybean. They are divided into two parts: 600 images for training and 600 images for testing. The FLO dataset^38^ contains 8189 images of 102 classes of flowers. The images are divided into 2040 training images and 6149 testing images from 102 classes. The CUB^9^ contains 5994 training images and 5794 testing images from 200 classes of birds. The CAR^10^ contains 8144 images for training and 8041 images for testing form 196 classes. The AIR^11^ contains 6667 training images and 3333 test images from 100 classes. For the PD^47^, 38 plant disease categories with 5700 training images and 5700 testing images are selected in this experiment.

We use VGG-16^1^ and ResNet-50^2^ as backbone network in our methods. The UFG operation^12^ is followed to keep the aspect ration of the original object shapes. In this operation, the input images are padded to square before being resized to the size of $440\times 440$ pixels, and then they are randomly rotated and cropped to $384\times 384$ pixels. 160 epoches are trained by all the methods, using stochastic gradient descent with a batch size of 16. At first, the learning rate is set as 0.001 and then decreases by a factor of 10 every 60 epochs. Moreover, during the experiments, the benchmark methods with carefully fine-turning are set according to the corresponding papers.

### Parameter settings

**Figure 4:**
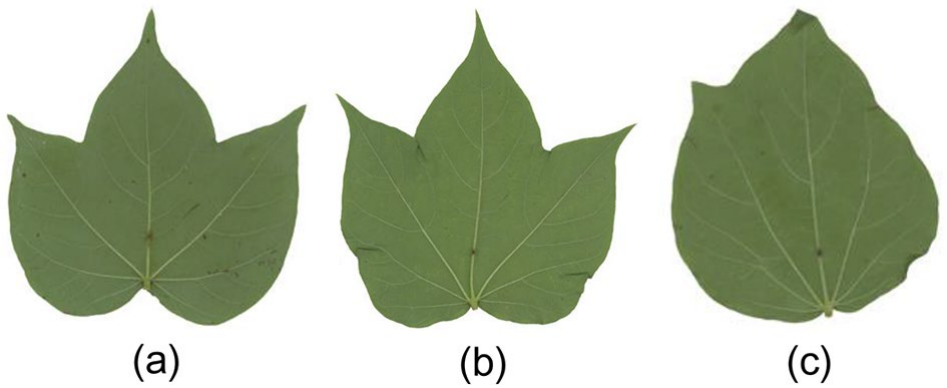
Example of different types of cotton leaf images.

Within the scope of our investigations, the UFG datasets^12^ is one of the most challenging datasets in FGVC. The reasons are as follows. The cotton and soybean image datasets include 80 and 200 very fine grained cultivars respectively, while they only have three training images in each category. On the other hand, their category attribution is mainly determined by genes, and it is difficult for human to accurately classify them. Take three cotton images as an example as illustrated in Fig. 4, it is easy for people to classify Fig. 4a and b into one category, and Fig. 4c in another category. In fact, Fig. 4b and c are of the same category, and Fig. 4a is from another category.

In this subsection, we discuss the selection of the number of sub-image blocks *N* and the image rotation directions. We first fix the input image set as $\{I,I_{\frac{\pi }{4}},S,S_{\frac{\pi }{4}}\}$ to check the accuracy of FGVC of the proposed method with different number of sub-image blocks. *I* represents the original image, $I_{\frac{\pi }{4}}$ represents the rotated original image counterclockwise by $\frac{\pi }{4}$, *S* represents the shuffled image, and $S_{\frac{\pi }{4}}$ represents the rotated shuffled image by rotating $\frac{\pi }{4}$ counterclockwise. It can be observed from Table  1 that the proposed method achieves the best performance when *N* is 6.

**Table 1:** Accuracy of the proposed method.

The number of sub-image blocks	$N=1$	$N=2$	$N=4$	$N=6$	$N=8$
Accuracy (%)	54.41	58.53	59.23	59.70	58.95

**Table 2:** Comparison with the state-of-the-art methods on six different standard datasets.

Method	Base Model	Accuracy (%)						
		Cotton	Soybean	CUB	CAR	AIR	FLO	PD
ResNet-50^2^	ResNet-50	52.17	39.83	84.20	90.92	89.74	95.35	96.33
VGG-16^1^	VGG-16	49.80	38.46	82.18	87.55	90.32	94.37	–
NTS-Net^4^	ResNet-50	51.30	43.80	84.23	90.32	88.15	95.42	–
fast-MPN-Cov^3^	ResNet-50	49.85	38.35	85.12	88.61	90.26	96.33	–
DCL^5^	ResNet-50	53.92	46.03	85.47	92.18	90.58	96.49	–
Cross-X^6^	ResNet-50	50.83	43.56	85.22	92.18	89.84	96.12	93.63
MOMN^8^	ResNet-50	43.34	37.58	81.79	86.25	85.33	97.15	98.58
ACNet^22^	ResNet-50	55.32	51.60	85.31	**92.29**	88.65	96.88	–
Ours	VGG-16	53.24	46.60	84.20	91.06	88.52	96.62	–
Ours	ResNet-50	**60.83**	**53.67**	**85.78**	**92.29**	**90.88**	**97.16**	**98.88**

Significant values are in [bold].

**Figure 5:**
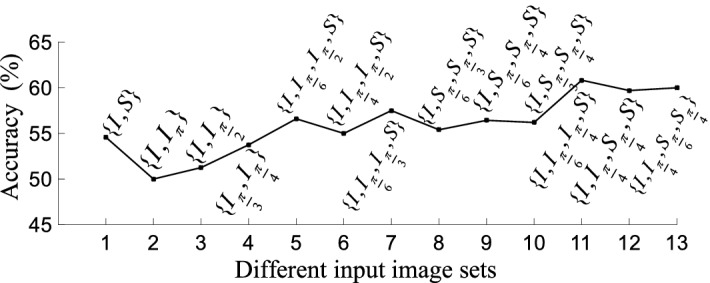
The impact of different input image sets on FGVC performance.

Secondly, we fix the number of the sub-image blocks *N* to 6 to check the accuracy of the proposed method with different input image sets. Figure 5 indicates that the FGVC performance is greatly impacted by the numbers of image rotations in different directions. It can be seen in Fig. 5 that, the performance of the image sets with 4 images is better than that of the image set with 2 images. Moreover, the proposed method has the best performance with image set $\{I,I_{\frac{\pi }{6}},I_{\frac{\pi }{4}},S\}$ and the worst performance with image set $\{I,I_{\pi }\}$, as shown in Fig. 5. On one hand,the input images of the image set with 2 images are *I* and $I_{\pi }$, which provide no innovation but the same LSI to the network (see Fig. 2). On the other hand, the input images of the image sets with 4 images have different LSIs and thus provide more information to the network. This is the reason for the results in Fig. 5.

Considering the results in Table 1 and Fig. 5, we set the sub-image blocks number to $N=6$ and the input image set to $\{I,I_{\frac{\pi }{6}},I_{\frac{\pi }{4}},S\}$ in the proposed method for subsequent experiments.

### Experiment results

Table 2 shows the direct results of our proposed method and the eight state-of-the-art methods on the six standard datasets. However, there are 7 datasets in Table 2, because the UFG datasets includes a soybean dataset and a cotton dataset. Moreover, we use our proposed method with the backbone of ResNet-50 as statical test to compare the examined methods. For CUB dataset, our proposed method achieves 1.58%, 3.6%, 1.55%, 0.66%, 0.31%, 0.56%, 3.99% , and 0.47% improvements over ResNet-50^2^, VGG-16^1^, NTS-Net^4^, fast-MPN-Cov^3^, DCL^5^, Cross-X^6^, MOMN^8^, and ACNet^22^; for CAR dataset, our proposed method achieves 1.37%, 4.74%, 1.97%, 3.68% , 0.11%, 0.11%, and 6.04% improvements over ResNet-50^2^, VGG-16^1^, NTS-Net^4^, fast-MPN-Cov^3^, DCL^5^, Cross-X^6^, and MOMN^8^, and similar accuracy as ACNet^22^; for AIR dataset, our proposed method achieves 1.14% , 0.56%, 2.73%, 0.62%, 0.30%, 1.04%, 5.55%, and 2.23% improvements over ResNet-50^2^, VGG-16^1^, NTS-Net^4^, fast-MPN-Cov^3^, DCL^5^, Cross-X^6^, MOMN^8^, and ACNet^22^; for FLO dataset, our proposed method achieves 1.81%, 2.79%, 1.74%, 0.83%, 0.67%, 1.04%, 0.01%, and 0.28% improvements over ResNet-50^2^, VGG-16^1^, NTS-Net^4^, fast-MPN-Cov^3^, DCL^5^, Cross-X^6^, MOMN^8^, and ACNet^22^. Table 2 indicates that the performance of our proposed method is better than that of the benchmark methods. The reason is that the network can learn more LSI of feature from each input image by using our proposed method. In other words, our proposed method can better depict the properties of different features in images. Furthermore, it can be observed from Table 2 that our proposed method achieves far better performance on datasets with a limited number of images such as the cotton and soybean datasets. The reason is that the accurate extraction of LSI of different features in images has a more significant impact on the performance of FGVC in a dataset with a limited number of images.

**Figure 6:**
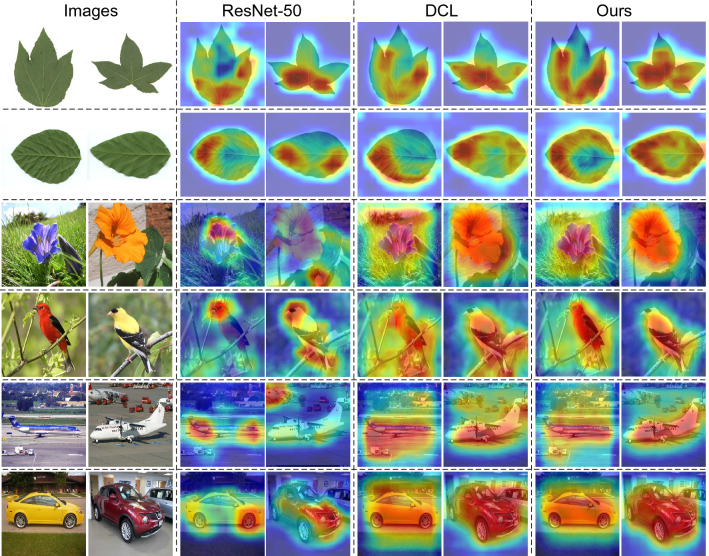
Feature map visualization of our method and two other methods based on the last convolution layer of ResNet-50 backbone.

**Figure 7:**
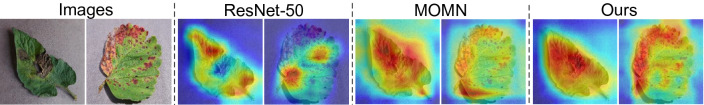
Feature map visualization of our method and two other methods based on the last convolution layer of ResNet-50 backbone.

For UGG, CUB, CAR, AIR, and FLO images, their corresponding feature maps of the last convolution layer of our method and two benchmark methods (ResNet-50^2^ and DCL^5^) are shown in Fig. 6. For PD images, their corresponding feature maps of the last convolution layer of our method and two benchmark methods (ResNet-50^2^ and MOMN^8^)are shown in Fig. 6. It can be seen from Figs. 6 and 7 that the feature maps of each method has a significant difference. Compared with the three other benchmark methods, our method concentrates on learn the overall structure information of the objects. Therefore, the interference of the surrounding environment on FGVC can be effectively suppressed.

The results in Table 2, Figs. 6 and 7 indicate that our proposed method has better performance than the existed methods. The main reason is that the proposed method can obtain the complete local structural features from input images by extracting LSI along multiple filter orientations. By this way, the sufficient LSI of each input image can be used for analyzing the properties of different salient regions and performing more effective FGVC. In other words, adding the proposed LSI learning module into a given backbone network can enhance the ability of the network to find salient regions.

Furthermore, we report our inference time on a NVIDIA Geforce GTX TITAN with PyTorch implementation. The running time on an image of size $384 \times 384$ is about 31 ms which means that our proposed method is computationally efficient in practical applications.

## Conclusion

In this paper, a novel LSI learning framework is proposed for FGVC. Firstly, the way of accurately extracting LSI from each input image is illustrated for the network to properly describe the properties of different features in images. Secondly, our framework for LSI learning is proposed. Thirdly, the performance of our proposed method is compared to that of the eight benchmark methods. Simulation results show that our proposed method has better ability in FGVC. Particularly, our proposed method has much better performance in dealing with the datasets with a limited number of images. It is worth to note that our proposed LSI learning mechanism has no obvious performance advantage when used for image object detection. In the following, we will extend the proposed mechanism to transformer^48^ and apply it for other image processing tasks such as object detection, image segmentation, and object tracking.

## Data Availability

The code that supports the results within this paper is not publicly available due commercial application in surface defect inspection but are available from the corresponding author on reasonable request.
